# Conceptual Framework for Nutritional Psychology as a New Field of Research

**DOI:** 10.3390/bs15081007

**Published:** 2025-07-24

**Authors:** Nanette Stroebele-Benschop, Vladimir Hedrih, Shereen Behairy, Nabila Pervaiz, Ephi Morphew-Lu

**Affiliations:** 1 Department of Applied Nutritional Psychology, University of Hohenheim, Fruwirthstraße 12, 70599 Stuttgart, Germany; 2 Faculty of Philosophy, University of Niš, Ćirila i Metodija 2, 18000 Niš, Serbia; vladimir.hedrih@filfak.ni.ac.rs; 3 Center for Nutritional Psychology, 467 Saratoga Ave, No 173, San José, CA 95129, USA; shereenbehairy@nutritional-psychology.org (S.B.); nabilapervaiz17@gmail.com (N.P.); ephilu@nutritional-psychology.org (E.M.-L.)

**Keywords:** nutritional psychology, conceptual framework, nutrition, psychology

## Abstract

Many recent discoveries highlight the existence of a robust bidirectional link between nutrition and psychological processes. Despite these developments, the systematic and formalized study of this connection is only beginning to be undertaken, and nutritional psychology is not yet considered a formal area of study within the psychological sciences. This paper defines the scope of nutritional psychology through 6 core areas of conceptualization, each informed by an interdisciplinary and growing body of evidence spanning the psychological and nutritional sciences. These include the diet-conative/affective, diet-cognitive, diet-sensory/perception, diet-interoceptive, diet-psychosocial, and diet-environmental relationships. Introducing these conceptualizations contributes to the development of innovative interdisciplinary language, method, and conceptualization of the diet-mental health relationship within nutritional psychology.

## 1. Introduction

Upon careful examination, one can see that the intricate relationship between nutrition and mental health spans the pages of history, reflecting a profound intuitive awareness of the connection between dietary practices and psychological well-being (Kleisiaris et al., 2014; Ross & Margolis, 2018). This foundational understanding has reverberated through the centuries, with medieval scholars and Renaissance thinkers recognizing the influence of nutrition on mental states (Dell’Osso et al., 2016; Kroll & Bachrach, 1984). Their insights have contributed to a holistic view of health, encompassing both the physical and psychological aspects of well-being (R. H. Li, 2022).

While the connection between nutrition and physical health has long been explicitly recognized, the evidence-based understanding of the impact of our diet on human behavior in general and psychological health and emotional well-being in particular has recently garnered significant attention from scientists, healthcare professionals, and the general public alike (Firth et al., 2020; Mueller-Stierlin et al., 2022). What we eat, how, where, and when we eat can substantially change how we think, feel, act, and experience. In turn, how we think, feel, and experience certain situations can change what we eat, how much we eat, or how the food tastes. Furthermore, rapid globalization and evolving dietary habits, accompanied by the worldwide increase in mental health disorders, have introduced a pressing need to explore innovative and comprehensive approaches to enhancing psychological well-being (Godos et al., 2020).

**Figure 1:**
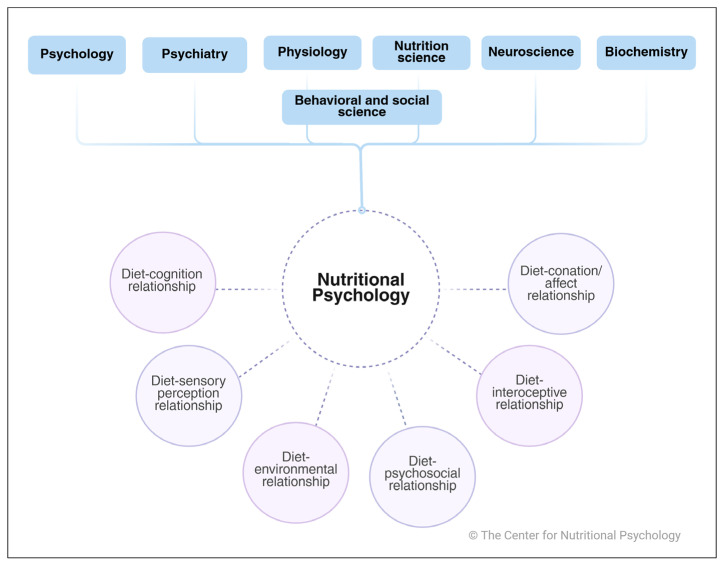
Scientific disciplines and core domains are incorporated into the framework of nutritional psychology.

To better understand abnormal eating behavior, foster stable mental health, and find ways to prevent and treat abnormal behavior and unstable mental health, it is crucial to look at everyday behavior first. Nutritional psychology (NP) uses psychological theories and approaches to explain, understand, and investigate general eating behavior (as well as abnormal eating behavior). The field of Nutritional Psychology (NP) encompasses various aspects of how our dietary choices impact and are impacted by behavior, cognitive function, emotional states, and even the development of mental health disorders. NP emerges as an innovative interdisciplinary field situated at the dynamic crossroads of nutrition and psychology; it synthesizes insights from various disciplines, including the psychological, behavioral, and nutritional sciences, neuroscience, neurobiology, physiology, and psychiatry (Figure 1). Years of mapping, exploring, and identifying the existing body of literature at the intersection of nutrition and psychology have led to the following definition of NP proposed by the Center for Nutritional Psychology (CNP) (Center for Nutritional Psychology [CNP], 2025a) and its classification into six core areas.

NP explores the relationship between nutrition and various psychological processes, including cognition, emotion, behavior, psychosocial functioning, sensory perception, interoception (encompassing both body and brain), and mental health. We define nutritional psychology as *a scientific discipline that studies the bidirectional relationships between nutrition and all psychological processes involving the mind*, *including cognition*, *emotions*, *behavior*, *psychosocial functioning*, *sensory perception*, *interoceptive experience (body and brain)*, *and mental health.* Recognizing the multidimensional nature of these relationships, NP examines how nutrition influences psychological processes, how psychological states influence nutrition-related behaviors, and the factors that shape both. Scientifically, NP bridges theories and findings from multiple classical disciplines.

## 2. Historical Background

While interest in the connection between nutrition and psychology began to emerge in the 1980s, approaches and studies concerning human nutrition and its influence on psychiatric disorders occurred before this (what is now referred to as nutritional psychiatry). Nutritional psychiatry focuses on the clinical application of dietary interventions for mental health disorders. Research in nutritional psychiatry investigates the causal connection between diet and psychiatric disorders to support the development of nutrition-based clinical interventions in mental healthcare (Adan et al., 2019; Jacka et al., 2017). For instance, the SMILES trial was the first randomized controlled trial to demonstrate that participants who followed a modified Mediterranean diet experienced significant reductions in depression symptoms, establishing a causal link between diet and mental health (Jacka et al., 2017).

However, the first book to use the terms *psychology* and *food*, which could be seen as a popular science book rather than an academic textbook, was Bernard Lyman’s *A Psychology of Food: More than a Matter of Taste* (Lyman, 1989). In the 1990s and 2000s, several books were published on the connection between food, eating, and psychology, though most were not formal textbooks (Booth, 1994; Logue, 2014; Ogden, 2010; Wardle, 1993). A glance at their content reveals differing perspectives on eating and behavior. Lyman, for example, focused on taste preferences and the influence of context, sensory aspects, or emotions. Booth explored how sensory, physiological, cultural, and interpersonal factors influence food choices and food consumption, grounding his approach in learning theory and framing dietary decisions as learned stimulus-response interactions involving interoceptive, social, and environmental inputs. In contrast, Ogden focused her book on disordered eating behavior and utilized various dietary decision-making models to explain the development and treatment of obesity, body dissatisfaction, and eating disorders. Blackman and Kvaska (2011) published Nutritional Psychology, the first book to systematically apply several subfields of psychology to dietary behavior, as outlined below. These included, for example, cognitive or emotional domains, as well as perspectives from perceptual psychology. Furthermore, the authors integrated various psychological approaches, including evolutionary biological, psychoanalytic, biopsychological, and behaviorist perspectives, to explain eating behavior.

To the authors’ knowledge, there is no existing framework around Nutritional Behavior, although it overlaps with many areas now encompassed by NP. The term *Behavioral Nutrition* was first introduced by Oltersdorf in 1984, who defined it as “the sum of all planned, spontaneous or habitual actions by individuals or social groups to procure, prepare and consume food as well as those related to storage and clearance” (Oltersdorf, 1984). Within the field of Behavioral Nutrition, there are four dimensions mentioned: health, environment, economy, and society (K. Schneider & Hoffmann, 2011). By contrast, NP also integrates psychological dimensions, such as emotions, cognitions, and interoception, that go beyond behavioral and societal influences alone. Nevertheless, the International Society for Behavioral Nutrition and Physical Activity (founded in 2000) was the first international multidisciplinary professional society that brought together researchers interested in behavioral aspects of nutrition (and physical activity).

These efforts, along with a growing body of scientific studies, represent early attempts to integrate nutrition with the psychological sciences. However, at the time, these were not grounded in a cohesive evidence base or unifying theoretical model. Only recently has the connection between nutrition and psychology gained recognition as a formal topic within psychological inquiry, requiring the development of a structured framework, methodology, and interdisciplinary language.

## 3. Methodology (Developing Nutritional Psychology as a Conceptual Framework)

The idea that nutrition and psychological processes are linked has been present in science for a long time. As time went on, the conceptual framework for this field has been evolving, and CNP has significantly accelerated this work since its establishment in 2015. The following section outlines the methodological steps used to construct the conceptual framework of NP, following the procedures for conceptual framework development by Jabareen (2009).

### 3.1. Mapping the Selected Data Sources

Since its founding, CNP has been tracking and compiling scientific publications that explore the connections between nutrition and psychological processes. It continuously monitors publicly available scientific publishing platforms and databases, primarily those publishing in English, for publications related to these topics.

### 3.2. Reading and Categorizing of the Selected Data

The publications are reviewed by CNP staff, a multidisciplinary team comprising mental health and nutrition-related professionals, researchers, professors, and educators. During this work, it has been noted that scientific outlets publishing works on the topic of nutritional psychology are mainly those from psychological, behavioral, and social sciences, nutrition sciences, neurosciences, biochemistry, physiology, and psychiatry.

CNP then curates and classifies the most representative publications into its research library and presents them to the public through its science publication (Center for Nutritional Psychology [CNP], 2025c). Currently, the CNP research library comprises over 3000 scientific publications relevant to the field of NP.

### 3.3. Identifying and Naming Concepts

Through this work, key concepts belonging to nutritional psychology and related to it have been extracted from these publications and included in the Encyclopedia of Nutritional Psychology maintained by CNP (Center for Nutritional Psychology [CNP], 2025b).

### 3.4. Deconstructing and Categorizing Concepts

A further aim was to organize these concepts and the studies in which they appear into areas of research, to support the development of a definition for the discipline of nutritional psychology.

### 3.5. Integrating Concepts

Upon inspecting the publications, an initial grouping of key concepts and topics of inquiry was made into five relationships that defined the broad areas of research in nutritional psychology.

### 3.6. Synthesis and Resynthesis

The authors of this study reviewed the division of concepts and nutritional psychology topics of inquiry into the five areas and have decided to reconceptualize them, reduce overlap, and divide concepts and topics of inquiry that deal with psychological characteristics using the classic division of psychological processes into cognitive, conative, and affective. In this way, the current definition of the six areas of nutritional psychology has been produced.

### 3.7. Validating the Conceptual Framework

The current phase of development involves presenting this conceptual framework to the scientific community for validation through peer-reviewed publication. It is anticipated that this presentation will initiate scholarly dialogue, facilitate critique, and support future refinements that advance the formalization of NP as a scientific discipline.

## 4. Results (Six Core Areas of Nutritional Psychology)

A body of evidence is rapidly emerging to deepen our understanding of how nutrition and psychology interconnect. Findings from numerous disciplines touching on this intersection are being identified, categorized, and consolidated in an online global repository, the Nutritional Psychology Research Library (Center for Nutritional Psychology [CNP], 2025c). These efforts, which build on the methodological framework outlined in the Methodology section, are leading to the development of a new interdisciplinary language, methodology, and conceptualization to better understand the connection between nutritional intake and psychological processes, experience, and outcomes.

**Figure 2:**
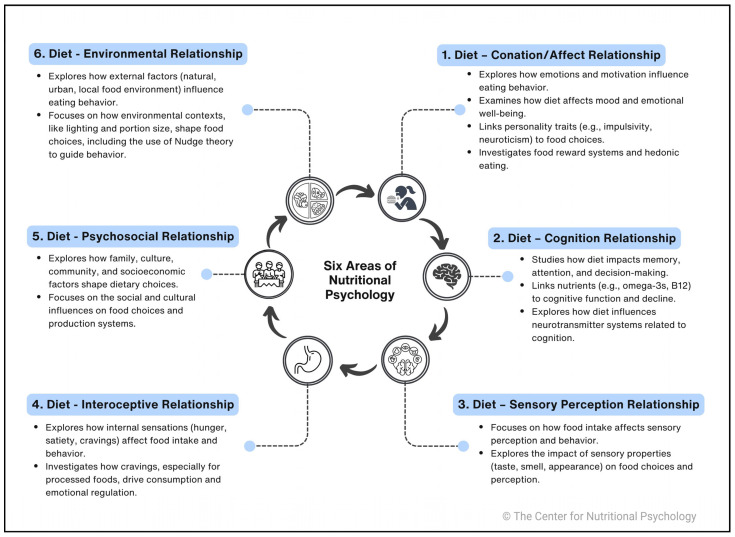
Example of content within the six core areas of nutritional psychology.

To better structure a conceptual approach to nutritional psychology, the authors propose six core areas of inquiry, each illustrating a distinct aspect of the diet-psychology relationship. We suggest that these six core areas should form the basis for conceptualizing nutritional psychology as a field of study. For each area, we will summarize key findings and identify critical studies to offer a comprehensive review. Figure 2 shows examples of topics within the six core areas of nutritional psychology.

**A. Diet—Conation/Affect Relationship:** Within this area, the bidirectional relationship between food intake patterns and motivational and emotional processes, including studies linking dietary choices to personality traits and reward processing, is examined and elaborated.

The relationship between emotions and food intake has been extensively researched (Chawner & Filippetti, 2024; González et al., 2022; Macht, 2008) with a particular focus on emotional eating and its relation to health outcomes such as weight gain, overweight, and eating disorders (Devonport et al., 2017; Godet et al., 2022). An abundance of research studies shows that negative emotions can lead people to eat more, in particular more of unhealthy foods high in fat, sugar, and salt, but also positive emotions result in increased eating (Evers et al., 2018). Eating for pleasure (i.e., hedonic eating) can enhance mood, while feeling down or depressed can negatively impact both appetite and taste perception (e.g., Bédard et al., 2020; Espel-Huynh et al., 2018; Ha & Lim, 2023).

But not only can mood affect food intake and food choice, research has also shown that food influences mood. Different foods and nutrients can have an emotional impact (AlAmmar et al., 2020). The most often used example is the effect of chocolate consumption on mood (Parker et al., 2006). Various studies have demonstrated an improvement in mood with dietary interventions (Bayes et al., 2022; Francis et al., 2019; Jacka et al., 2017; O’Neill et al., 2022), and dietary behavior seems to be associated with subjective well-being and happiness (e.g., De Leon et al., 2022; Ocean et al., 2019). On a more immediate/short-term level, studies have indicated that feelings of hunger (that cannot be immediately satiated by eating desired food) can make a person more likely to experience anger and other negative emotions, giving rise to the concept of being “hangry” (Swami et al., 2022). Thus, the bidirectional relationship is important when investigating and elaborating on the diet—conative/affective relationship (AlAmmar et al., 2020).

Additionally, the relationship between diet and personality factors is part of this core area. Personality factors are described as enduring characteristics and behavioral traits that comprise a person’s unique self. It includes major traits, interests, drives, values, abilities, and emotional patterns (American Psychological Association (APA), 2025). Research indicates that different personality traits are linked with different food preferences, food choices, and favorable and unfavorable eating patterns (Hampson et al., 2015; Z. Li et al., 2025; Pristyna et al., 2022; Spence, 2021). For instance, openness and extraversion seem to be positively associated with recommended and non-recommended foods, whereas neuroticism seems to be positively associated with non-recommended foods (Pristyna et al., 2022). Studies have also linked traits like impulsivity or behavior inhibition system activity (from Gray’s reinforcement sensitivity theory) to experiences of food cravings and food addiction (e.g., Loxton, 2018; Maxwell et al., 2020; Nederkoorn et al., 2006).

A third construct within the diet and conation/affect relationship is food reward processing and motivation to eat. Mainly, this research is focused on understanding the drivers and mechanisms of overconsumption using the assumption that today’s food consumption is not driven by homeostatic needs but rather by the rewarding aspects of the foods consumed (Berthoud et al., 2011). Since our reward system is located in the brain, conducting human research in this area is difficult and complex. Nevertheless, studies found associations between neuro-adaptive responses in brain reward circuits and the development of obesity as well as compulsive eating habits (Chen et al., 2008; Martin et al., 2010; Simon et al., 2014; Stice et al., 2010), indicating abnormalities in the reward system in people with obesity (Gill et al., 2021; Oustric et al., 2018). These links have much more extensively been explored in rodents, with researchers pointing to separate neural pathways reactive to fats and to sugars leading to reward the processing areas of the brain as key for understanding the strong rewarding effects of foods rich in easily digestible fats and sugars, such as modern ultra-processed foods (Lippert et al., 2020; McDougle et al., 2024). Studies also identified neural circuits (in rodents) that initiate homeostatic feeding and suppress hedonic feeding (Atasoy et al., 2012; Liu et al., 2024).

**B. Diet—Cognition Relationship:** Within this core area, the investigation of the effects of dietary patterns on cognitive processes can be found, with studies exploring bidirectional links between nutrition and memory, attitudes, attention, and decision-making (Clemente-Suárez et al., 2025). Most prominent is the research around nutrients such as omega-3 fatty acids or vitamin B12 and their impact on cognitive functions, cognitive decline, and neurodegenerative diseases (Kalamara et al., 2025; Lauer et al., 2022; Rogers et al., 2008). In general, dietary intake influences neurotransmitter systems, and disruptions in neurotransmitter balance due to dietary factors can impact both cognitive and emotional health. Additionally, research has linked dietary patterns such as the Western-style diet to cognitive decline and neurodevelopmental disorders, including Attention Deficit Hyperactivity Disorder (ADHD) (e.g., Chou et al., 2018; Howard et al., 2011; Pinto et al., 2022). In contrast, the Mediterranean diet is associated with enhanced cognitive outcomes and a lower risk of mental health disorders such as depression and anxiety (e.g., Camprodon-Boadas et al., 2024). Generally, highly palatable foods negatively impact cognitive function (e.g., Stevenson et al., 2020).

However, studies looking at the other direction, looking at the influence of our memories and attitudes, reveal the influence of both on our food choices. For example, a 2022 review reports on studies showing that amnesia is associated with impaired processing of hunger and satiety cues, disrupted memory of recent meals, and overconsumption (Parent et al., 2022). On the other hand, meal-related memories of healthy participants limit subsequent ingestive behaviors. Collectively, studies presented in that review indicate that diet-induced obesity may be caused or maintained, at least in part, by a vicious cycle wherein excess food intake disrupts the functioning of the hippocampus region of the brain related to memory, further increasing intake. Other studies have shown that our attitudes towards animal welfare or the environment shape our food choices substantially (Janssen et al., 2016; Sanchez-Sabate & Sabaté, 2019; Testa et al., 2021). Furthermore, memories of previous eating situations can shape future food choices, even when they are false memories (Bernstein et al., 2005; Laney et al., 2008). Visualizing or remembering a recent meal can influence subsequent food intake (e.g., Higgs, 2008; Szypula et al., 2023), and the amount of attention paid to food consumption can influence its taste, the amount consumed, and many more aspects of a meal (e.g., Higgs, 2015). Moreover, the conscious decision to restrain food intake significantly impacts eating behavior and is often more mentally demanding for individuals with obesity (e.g., Wang et al., 2020). Restrained eating involves the deliberate attempt to control food intake for weight management or weight loss by setting self-imposed limits and regulating eating patterns. Cognitive restraint, a component of restrained eating, refers to the intentional mental effort to restrict food intake through internal rules or goals. It is often associated with emotional and psychological challenges, particularly when lapses occur. Such deviations from self-imposed boundaries can lead to negative emotional states and may contribute to overeating due to the stress or guilt associated with breaking these rules (e.g., Verzijl et al., 2018).

**C. Diet—Sensory Perception Relationship:** Exploring how food intake patterns affect sensory-perceptual processes, focusing on the influence of food-related stimuli on behavior and the impact of food intake on environmental perception (Y. Li et al., 2025) constitutes this area within nutritional psychology. Again, this relationship can be described as bidirectional. The sensory properties of the food (taste, smell, look) can determine whether an individual will decide to try it or consume it, and our prior experiences with food can affect how we perceive certain foods. For example, multiple studies indicate that repeatedly exposing toddlers to food (vegetables) they initially disliked while having no experience with them will make most of them accept and readily eat them (Elmas & Kabaran, 2023; Maier et al., 2007). Our senses, such as sight, sound, smell, taste, flavor, texture, haptics, and palatability, collectively influence food preferences, perceptions, choices, and overall dietary intake patterns. Extensive research has been conducted on this relationship (e.g., Hoppu et al., 2021), notably by Charles Spence and Betina Piqueras-Fiszman (Spence & Piqueras-Fiszman, 2014). While the influence of the taste and smell of food is clearly connected to consumed amounts, the effects of the color or texture of food seem to be less straightforward. For instance, even when flavor remains constant, presenting the same food in multiple colors can increase consumption (Kahn & Wansink, 2004). Similarly, the shape of dishware can alter food perception (e.g., Clarke et al., 2021; Langfield et al., 2020; McClain et al., 2014). These sensory factors influence eating behavior before, during, and after consumption and can be triggered by mere thoughts of food. Moreover, certain foods can affect the perception and taste of other foods. An example is sensory-specific satiety, which refers to the reduced pleasantness of a food as it is consumed relative to an uneaten food (e.g., Wilkinson & Brunstrom, 2016). This phenomenon drives dietary variety and helps explain why individuals might have room for dessert after a substantial meal (e.g., Smeets et al., 2010).

The perception of a meal or food can also affect taste. For instance, the same meal rated in a restaurant is often perceived as more enjoyable compared to when it is served in a student cafeteria, highlighting how location and expectations influence taste (Meiselman et al., 2000; Wu et al., 2022). Additionally, the labeling of food products can impact physiological responses; an experiment by Park et al. (2020) demonstrated that labeling a drink as “high sugar” can lead to an increase in blood sugar levels, even if the drink contains no actual sugar, illustrating how expectations can affect bodily reactions (Park et al., 2020). Differences in experience with certain foods can also alter which sensory aspects of a food are prioritized when making decisions about whether to consume certain foods or not. For example, while many find the idea of consuming insects repulsive, focusing on their visual aspects, the perceptions of individuals who were culturally exposed to insect meals seem to be primarily driven by their taste (Tan et al., 2015).

**D. Diet—Interoceptive Relationship:** The area within nutritional psychology described as diet-interoceptive relationship analyses the relationship between internal physiological sensations (e.g., hunger, satiety, post-ingestive signals) and psychological experiences, including the microbiota-gut-brain axis (Davidson & Stevenson, 2022; Stevenson, 2024).

The diet-interoceptive relationship encompasses the internal bodily sensations experienced in relation to food intake. In NP, these interoceptive sensations include hunger, thirst, satiety, post-ingestive sensations, and the palatability of food, all of which can lead to consumption or reduce consumption. These sensations interact with psychological aspects of nutrition, shaping eating patterns. In the absence of cognitive restraint, hunger, thirst, and high palatability of presented food generally drive consumption, while feelings of satiety and fullness typically signal the termination of intake.

One aspect of this relationship is the neurobiological process of cravings. Cravings, often associated with specific, typically savory foods, can trigger intense interoceptive sensations that drive individuals to seek and consume the craved food. These cravings are not solely prompted by the presence of food stimuli; they can also be spontaneously triggered by mere thoughts of the desired food (Richard et al., 2017). The satisfaction of food cravings is frequently perceived as providing short-term pleasure, stress relief, and emotion regulation. Notably, modern societies often associate cravings with highly processed foods, which are linked to less healthy dietary patterns (e.g., Massicotte et al., 2019).

While studies on hunger and thirst go back to the early nineteen hundreds (Cannon & Washburn, 1912), lately research on the bidirectional communication between the central and the enteric nervous systems—the gut-brain axis—has shown amazing links between the brain’s emotional and cognitive areas with intestinal functions (e.g., Carabotti et al., 2015). Recent research provides strong evidence that the diet influences gut microbiota and, with it, also brain functions (Oriach et al., 2016; Romaní-Pérez et al., 2021). For example, the typical Western diet appears to result in a loss of several bacterial species, followed by a reduction in microbial diversity and stability (Schnorr et al., 2014). Another study, for example, found that eating steamed broccoli sprouts alleviates gut lining inflammation, protecting the richness of gut microbiota communities (Holman et al., 2023). Conversely, stress, and particularly chronic stress, activates the hypothalamic-pituitary-adrenal (HPA) axis, leading to elevated stress hormones, which consecutively influence the activities of intestinal cells, often adversely affecting gut microbiota composition (e.g., Carabotti et al., 2015). This complex interaction plays a substantial role in regulating food intake and overall dietary behavior.

While classic views of hunger have primarily seen it as a body’s indicator of energy deficit, a sort of internal fuel gauge, new findings indicate that internal states humans interpret as hunger are quite diverse and that these interpretations are learned in childhood (Stevenson et al., 2023). According to this view, individuals learn which internal signals to interpret as hunger from their caregivers, as evidenced by the similarity between offspring and their primary caregivers in their beliefs about internal hunger states.

**E. Diet—Psychosocial Relationship:** Psychosocial factors—such as family setting, cultural background, community, society, and socioeconomic circumstances—are powerful influencing aspects regarding our dietary patterns. Lately, the effect of social and cultural influences on food choices has even caught the attention of agricultural and economic scientists, accepting the crucial impact of these influences on entire food (production) systems (Enriquez & Archila-Godinez, 2022; Hampton & Whitmarsh, 2023).

Where, how, and with whom one grows up substantially influences food choices and behaviors (Braune et al., 2024; Fernqvist et al., 2024). Social norms and cultural circumstances within a community or society also shape dietary patterns. A good example is the handling of alcohol in social contexts and its effect on youth consumption (Trucco et al., 2014). Different factors within the diet-psychosocial relationship can impact food choices, and certain food choices and dietary behaviors can even shape the psychosocial environment. For instance, research indicates that upbringing and family practices regarding food and beverages, such as the availability of soft drinks in the home, have a long-lasting impact on future dietary habits (S. Schneider et al., 2021). Additionally, early exposure to specific foods shapes preferences; for example, children in Mexican households tend to develop a preference for spicier foods compared to those in non-Mexican households in the U.S. (Siebert et al., 2022). Individuals culturally exposed to dishes that most people find repulsive, such as insects or fermented fish, find them acceptable, include them in their diets, and even consider them an important part of their cultural identity (Nygaard, 2019; Tan et al., 2015). On the other hand, experiencing chronic discrimination and high levels of unfair treatment even seem to be associated with an increased willingness to eat unhealthy foods (Zhang et al., 2023).

Another included aspect within the diet-psychosocial relationship is when social environments exert direct effects on eating behavior, as demonstrated by the social facilitation effect, which describes how the quantity of food consumed is influenced by the number of people present during meals (e.g., Herman, 2015). Another direct effect is the social modeling effect, wherein individuals adjust their food intake based on the eating behaviors of those around them (e.g., Vartanian et al., 2015). Parental and peer modeling has been shown to significantly influence both healthy and unhealthy eating behaviors in children and adolescents (e.g., Bassul et al., 2020; Romero et al., 2009). A recent review concludes that social modeling of eating occurs under various circumstances and across various populations (Suwalska & Bogdański, 2021). A 2013 study in China suggested that grandparents unequivocally accorded supreme importance to ensuring a child finishes their meals, while parents tended to be a bit more liberal (Goh, 2013). This author reports that the mentioned practice results in many children in multi-generational families being routinely force-fed.

Family dining rituals in many Asian cultures, such as the structured sharing and serving behaviors seen in traditional Chinese meals, reinforce collective norms that can inhibit restrained eating among individuals. These shared rituals, including serving elders first and communal dishes, create social expectations that influence what and how much one eats (Ma, 2015). On the other hand, manners such as pacing the meal, refusal strategies, and etiquette around leftovers may reinforce moderation and prevent overeating. In this way, eating behavior becomes a reflection of family identity, but also a reflection of one’s own identity.

Perceived social isolation or loneliness has also been associated with changes in eating behavior. A recent study found that individuals reporting higher levels of loneliness tend to exhibit poorer mental health, more disordered eating patterns, and lower-quality diets (Zhang et al., 2024).

**F. Diet—Environmental Relationship:** The diet-environmental relationship describes how external factors, such as the environmental contexts, including natural and constructed settings, influence eating behavior (Caspi et al., 2012; Glanz et al., 2005). The field of the study of external factors on eating behavior is huge, ranging from natural environments such as nature or urban environments (Langlois & Chandon, 2024; Méjean & Recchia, 2022) or more specifically the local food environment that gives people access to food (Caspi et al., 2012; Gesteiro et al., 2022; Glanz et al., 2005) to distinct factors within the environment such as the lighting environment (Meléndez-Fernández et al., 2023) while eating or the offered portion size (Higgins et al., 2021). Applications of the Nudge theory tell how elements of one’s environment can form a person’s choice architecture and can gently drive behavioral choices humans make, including choices of food (Arno & Thomas, 2016).

As examples, simply experiencing nature has a positive impact on food choices (Langlois & Chandon, 2024), while the context of dining, such as eating in a fast-food restaurant versus at home, significantly influences total caloric intake regardless of taste perception or personal attitude (An, 2016; Powell & Nguyen, 2013). More individual environmental conditions, including outdoor and indoor temperatures, have been associated with variations in food choices and intake. For example, indoor temperatures in England have been linked to different BMI levels, and a similar effect has been observed for ambient temperatures in Spain (Daly, 2014; Valdés et al., 2014). Additionally, a randomized controlled experiment demonstrated changes in calorie intake in response to variations in office temperature (Bernhard et al., 2015). Another explored environmental factor is lighting. The color of light can influence the perception of food and subsequent taste expectations. However, the effect of experimentally varying light brightness on eating behavior remains less clear. A pilot study found that bright light was associated with higher hunger ratings compared to dim light (AlBreiki et al., 2015). Generally, lighting appears to play a role in the perceived pleasantness of the eating environment (e.g., Bschaden et al., 2020).

Two extensively studied areas regarding the impact of external factors on our eating behavior are convenience and portion size. Food that is more accessible, easier to prepare, and consume is more likely to be eaten (e.g., Chandon & Wansink, 2002; Hollands et al., 2013). Furthermore, larger portion sizes tend to increase consumption (e.g., Vargas-Alvarez et al., 2021).

On a broader level, researchers have proposed the concepts of food deserts and food swamps, i.e., areas with limited access to affordable and nutritious food and areas saturated with unhealthy, high-calorie food options, respectively. Studies have linked living in both food deserts and food swamps with increased risks of obesity and other health issues (Cooksey-Stowers et al., 2017; Kelli et al., 2019).

It is also likely that individual interpretations of environmental factors mediate the relationship between the objective properties of the environment and dietary behavior, suggesting that the diet-environmental relationship may be more nuanced than currently understood.

## 5. Discussion and Conclusions

Nutrition and health are unequivocally linked, and so are nutrition, eating behavior, and psychological processes, experiences, and outcomes. One’s psychological state can affect what, where, and how much one eats, and eating affects one’s psychological well-being, whether in relation to mood, cognition, or perception (Polivy & Herman, 2005). To better organize the extensive field of nutritional psychology, the authors propose six core areas within nutritional psychology that collectively contribute to our understanding of the relationship between nutrition and psychology across various disciplines. This classification of six theoretical conceptualizations within nutritional psychology can expand the theoretical framework of nutritional psychology within the psychological sciences, enhancing conceptualization, methods, and universal terminology in the field, which will better help structure educational programs in nutritional psychology. Such programs are emerging across the world and increasingly becoming part of both university and continuing education of mental health professionals, such as psychologists, social workers, dietitians, nutritionists, psychiatrists, and others.

It is crucial to recognize that these areas are dynamically interrelated and do not occur in isolation; rather, in applied settings, elements of each area are present and interacting within each situation and experience. Each component, alone or in combination, can influence behavior or result in behavioral consequences. For example, an individual may regularly visit a restaurant that serves large portions of food. A large portion size tends to make the individual eat more. However, that individual might be with their significant other and might be motivated not to be perceived as a glutton. This might make the person eat less or choose leaner food despite the portion size or their preferences. However, such decisions might leave the person hungry, motivating them to take additional snacks during the evening or late at night. If situations such as this repeat, the person may form a habit of late-night snacking. Late-night snacking, on the other hand, is associated with weight gain (Gluck et al., 2008). If the individual had plans to lose weight, this new habit might derail them (Dashti et al., 2021). It might also worsen the individual’s cardiometabolic health markers (Bermingham et al., 2024) and alter reward processing mechanisms in the brain and gut, changing the person’s future dietary preferences (Ni et al., 2019; Thanarajah et al., 2023), demonstrating a complex interplay between dietary choices, psychosocial environment, and individual psychological characteristics.

The aim of this article is to present a conceptual framework of nutritional psychology to introduce to the research community for discussion, evaluation, and further development. Additionally, the future agenda for this field is to examine how these six core areas are integrated, connected, and related.

By providing an expanded conceptualization within the scientific field of nutritional psychology, a more robust theoretical foundation is created, thereby supporting the continued development of new language, methods, evidence utilization, and growth of this interdisciplinary emerging field.
